# Concrete Open-Wall Systems Wrapped with FRP under Torsional Loads

**DOI:** 10.3390/ma5112055

**Published:** 2012-10-25

**Authors:** Geminiano Mancusi, Luciano Feo, Valentino P. Berardi

**Affiliations:** Department of Civil Engineering, University of Salerno, Fisciano 84084, Italy; E-Mails: l.feo@unisa.it (L.F.); berardi@unisa.it (V.P.B.)

**Keywords:** structural plating, FRP, torsional loads

## Abstract

The static behavior of reinforced concrete (RC) beams plated with layers of fiber-reinforced composite material (FRP) is widely investigated in current literature, which deals with both its numerical modeling as well as experiments. Scientific interest in this topic is explained by the increasing widespread use of composite materials in retrofitting techniques, as well as the consolidation and upgrading of existing reinforced concrete elements to new service conditions. The effectiveness of these techniques is typically influenced by the debonding of the FRP at the interface with concrete, where the transfer of stresses occurs from one element (RC member) to the other (FRP strengthening). In fact, the activation of the well-known premature failure modes can be regarded as a consequence of high peak values of the interfacial interactions. Until now, typical applications of FRP structural plating have included cases of flexural or shear-flexural strengthening. Within this context, the present study aims at extending the investigation to the case of wall-systems with open cross-section under torsional loads. It includes the results of some numerical analyses carried out by means of a finite element approximation.

## 1. Introduction

Within many theoretical and experimental studies available in literature on the use of fiber-reinforced composite material (FRP) for the structural strengthening of reinforced concrete (RC) members [1,2,3,4,5,6,7], the case of torsional loads does not seem to have been exhaustively investigated, although it frequently occurs in civil engineering.

In fact, there are many examples of torsional strengthening on existing structural elements (e.g., on lateral beams of stairways, wall-systems around elevator holes of buildings and finally, bridge decks subject to highly eccentric loads).

Although traditional and innovative solutions [8] have been proposed, the role played by concrete wall systems seems to be promising also from the perspective of the seismic mitigation strategies.

Recently, theoretical, numerical and experimental analyses on the structural plating by the use of FRP materials have been carried out by the authors, accounting for many relevant features: the peak values of interfacial interactions [9], the creep behavior of the composite reinforcement [10,11,12], the shear deformability [13,14] and also the constructive details or anchoring devices able to prevent delamination [15]. These studies investigated the possibility of correctly predicting the mechanical behavior of RC elements strengthened by FRP via appropriate theoretical models which take into account many peculiarities.

In the case of torsional loads, however, the warping displacements of the cross-section cannot be assumed negligible and they have to be explicitly taken into account.

To this purpose, the authors have developed a mechanical model capable to predict the behavior of a strengthened concrete wall-system with open cross-section (U shape) subject to torsion. The model also accounts for the shear deformability due to its relevance in the case of the composite [14].

The present paper develops a case-study concerning the torsional strengthening of a concrete wall-system with open cross-section (U shape) and analyzes the local interactions between the concrete member and the FRP reinforcement.

## 2. Structural Model

**Figure 1:**
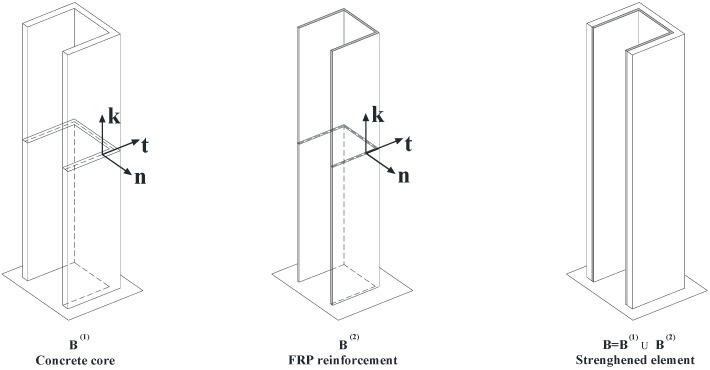
B^(1)^: Reinforced concrete (RC) member; B^(2)^: fiber-reinforced composite material (FRP) strengthening; $\text{B}={\text{B}}^{\left(1\right)}\cup {\text{B}}^{\left(2\right)}$: strengthened member.

Let us consider a U concrete wall-system strengthened with an external FRP laminate (Figure 1).

The response of the strengthened member (B) can be simulated accounting for the interaction of two one-dimensional beams which respectively represent the pre-existing RC member (B^(1)^) or the FRP strengthening (B^(2)^) (Figure 1).

In the following, the different aspects of the model will be summarized.

### 2.1. Kinematics

The kinematics proposed for each beam, B^(1)^ or B^(2)^, has been developed previously in [13,14].

Thus, the displacements components *ξ* , *η* and *ζ* of the single beam can be expressed as follows:

(1) $$\xi =\xi \;\left(s,z\right)=\;\xi_{\text{c}}\left(z\right)\;-\;\rho \;\left(z\right)\cdot \left(y(s)-y_{\text{c}}\right)$$

(2) $$\eta =\eta \;\left(s,z\right)=\;\eta_{\text{c}}\left(z\right)\;+\;\rho \left(z\right)\cdot \left(x(s)-x_{\text{c}}\right)$$

(3) $$\zeta =\zeta \left(s,z\right)=\;\zeta_{\text{c}}\left(z\right)\;-\;\beta \left(z\right)\cdot x(s)\;+\;\alpha \left(z\right)\cdot y(s)\;-\;\dot{\rho }\left(z\right)\cdot \omega \left(s\right)\;+\;\gamma_{\text{i}}\left(z\right)\cdot \omega_{\text{i}}\left(s\right)$$

For the sake of simplicity, in Equations (1–3) the subscript denoting which beam is referred to, has been omitted.

Furthermore, $x_{\text{c}}$, $y_{\text{c}}$ correspond to the coordinates of a fixed point C assumed as pole of a rigid transformation of B^(i)^ (i = 1, 2); $\xi_{\text{c}}\left(z\right)$, $\eta_{\text{c}}\left(z\right)$ are the displacement components of the point C along the *x* and *y* axes, respectively; α(z) and β(z) are the cross-section flexural rotations; $\zeta_{\text{c}}\left(z\right)=\zeta_{\text{M}}\;+\;\beta \left(z\right)\cdot x_{\text{M}}\;-\;\alpha \left(z\right)\cdot y_{\text{M}}$ with $\zeta_{\text{M}}={\zeta |}_{\text{s}=0}$ and $M(x_{\text{M}},\;y_{\text{M}})$ denotes the origin of the curvilinear abscissa introduced over the mid-line of the cross-section of B^(i)^; $\dot{\rho }\left(z\right)$ is the derivative of the twisting rotation ρ(z) with respect to the z coordinate, while ω(s) is the current sectorial area as in the classical theory of thin-walled beams.

Finally, $\gamma_{\text{i}}\left(z\right)$ and $\omega_{\text{i}}\left(z\right)$ (i = 1, 2, … , Ns) are, respectively, further generalized kinematical unknowns and generalized sectorial areas, as defined in [14]. The unknowns $\gamma_{\text{i}}\left(z\right)$ provide an increasingly refined modeling of the shear deformability as the parameter Ns increases. This seems to be very relevant for the beam B^(2)^ (composite overlay) due to its low shear moduli of elasticity.

**Figure 2:**
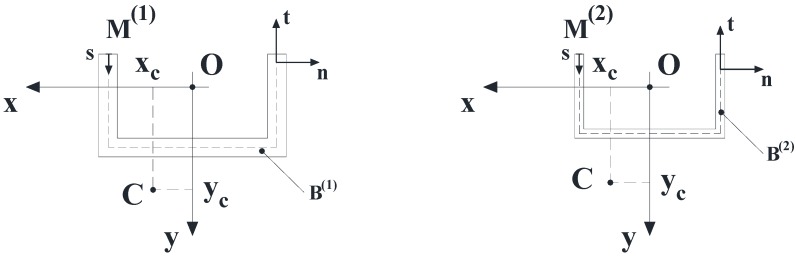
Cross-sections of B^(1)^ and B^(2)^.

The displacement field components (1–3) allow the generic cross-section of the component B^(i)^ (i = 1, 2) depicted in Figure 2 to exhibit: (i) A rigid transformation in its own plane; (ii) A warping out of the same plane; (iii) The following angular sliding along the mid-line:

(4) $${\gamma_{\text{tz}}\left(n,s,z\right)|}_{n=0}=\gamma_{\text{xz}}^{\left(0\right)}(z)\frac{dx}{ds}+\gamma_{\text{yz}}^{\left(0\right)}(z)\frac{dy}{ds}+\gamma_{\text{i}}\left(z\right)f_{\text{i}}\left(s\right)$$

where terms *f*_i_ (s) denote specific polynomials which have been defined in a general manner in [14].

The reader is invited to consult this previous work in order to understand in further detail the aspects of the kinematical model adopted.

### 2.2. Interface Model

Let Ω be the zero-thickness interface between B^(1)^ and B^(2)^; furthermore, let κ be the intersection between Ω and the plane that contains the current cross-section of $\text{B}={\text{B}}^{\left(1\right)}\cup {\text{B}}^{\left(2\right)}$.

**Figure 3:**
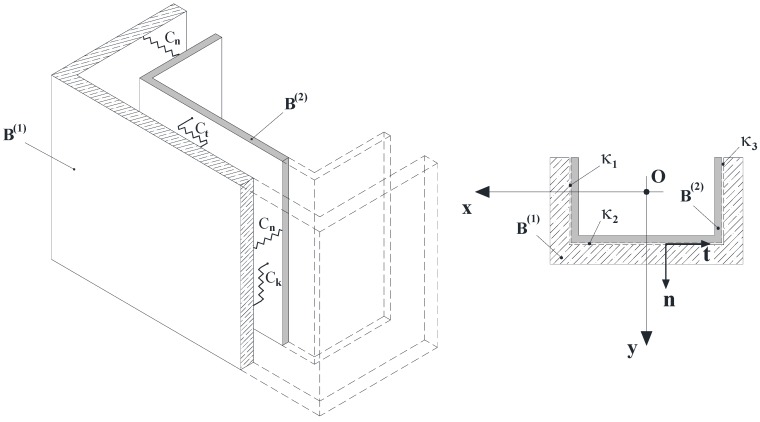
Interface between **B^(1)^** and **B^(2)^**.

It results $\text{Ω}\subseteq \partial {\text{B}}^{\left(1\right)}\cap \partial {\text{B}}^{\left(2\right)}$. It is also assumed that κ is composed of a finite number *N* of straight segments: ${\text{κ}}_{1}$, ${\text{κ}}_{2}$, … ,${\text{κ}}_{N}$ (Figure 3).

The model proposed by the authors assumes that the bonding between B^(1)^ and B^(2)^ is simulated by means of bilateral, independent elastic springs, which cover the entire surface Ω and are arranged continuously along the local axes {**n, t, k**}, the unit vector **n** being oriented from B^(2)^ to B^(1)^ [16,17].

It is worth denoting with *C*_n_, *C*_t_ and *C*_k_, in order, the compliance coefficients (per unit area) of the above mentioned springs along the directions **n, t** and **k**. The reactions of these interfacial springs ideally interposed between B^(1)^ and B^(2)^ are provided by the following relationships:

(5) $$t_{\text{n}}\;=\;\frac{1}{C_{\text{n}}}\left(u_{\text{n}}^{\left(1\right)}\;-\;u_{\text{n}}^{\left(2\right)}\right)$$

(6) $$t_{\text{t}}\;=\;\frac{1}{C_{\text{t}}}\left(u_{\text{t}}^{\left(1\right)}\;-\;u_{\text{t}}^{\left(2\right)}\right)$$

(7) $$t_{\text{k}}\;=\;\frac{1}{C_{\text{k}}}\left(u_{\text{k}}^{\left(1\right)}\;-\;u_{\text{k}}^{\left(2\right)}\right)$$

where $u_{\text{n}}^{\left(1\right)},\;u_{\text{t}}^{\left(1\right)}$ and $u_{\text{k}}^{\left(1\right)}$ indicate, in order, the displacements of a generic point of P∈Ω if it accords to the kinematics of the beam B^(1)^. They are expressed in the local reference system. Similarly, $u_{\text{n}}^{\left(2\right)},\;u_{\text{t}}^{\left(2\right)}$ and $u_{\text{k}}^{\left(2\right)}$ indicate the displacements of the same point P∈Ω if it accords, instead, to the kinematics of the beam B^(2)^.

The adhesion between B^(1)^ and B^(2)^ exhibits a progressively increasing stiffness as the quantities *C*_n_, *C*_t_ and *C*_k_ decrease. This provides an appropriate approximation of perfect contact interactions between B^(1)^ and B^(2)^ when the quantities *C*_n_, *C*_t_ and *C*_k_ tend towards zero.

### 2.3. Constitutive Assumptions

The concrete is assumed to be homogeneous, linear-elastic and isotropic; furthermore, no cracking occurs.

The presence of internal steel reinforcements is neglected due to the simplified constitutive law adopted for the concrete.

The composite strengthening is, however, assumed to be made of a homogeneous, linear-elastic orthotropic material.

Although the constitutive hypotheses of the reinforced concrete member have been simplified, the numerical analysis allows for evaluation of the feasibility of this type of strengthening in respect to the torsional loads, and provides an answer to the following question: is it possible to strengthen a concrete wall-system subject to torsional loads? If the system presents an open cross-section, the answer is affirmative, as discussed in the next section.

## 3. Case-Study 

**Figure 4:**
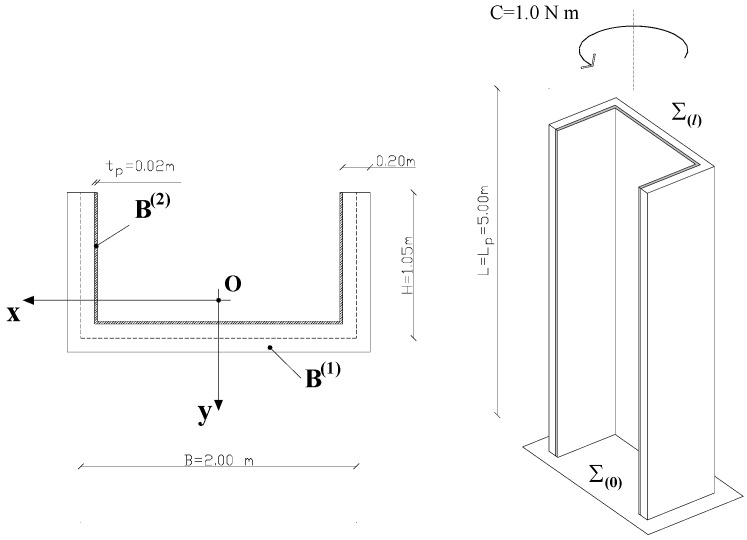
Details of the problem under consideration.

The example indicated in Figure 4, which concerns a concrete wall-system wrapped by a composite overlay, is intended to illustrate the benefits that can be obtained by restraining the warping of the composite at the bottom end of the scheme.

The numerical analyses have been performed via a finite element model previously introduced by the authors [9].

At the bottom end Σ_(0)_, all displacements for both the beam B^(1)^ and the beam B^(2)^ are constrained to be zero; warping is not allowed too. A unit torsional couple is applied on the free end Σ_(*l*)_ of the concrete wall-system.

The symbols used in Figure 4 have the following meaning: L: height of the concrete wall-system (L = 5,00 m); L_p_: length of the strengthened region (L_p_ = L); B, H: cross-sectional sizes of **B^(1)^** in reference to its mid-line; t_p_: overall thickness of the composite wrapping (t_p_ = 20 mm); Σ_(0)_, Σ_(*l*)_: bottom/top end; C: unit torsional couple applied on the free end Σ_(*l*)_ of **B^(1)^**.

**Figure 5:**
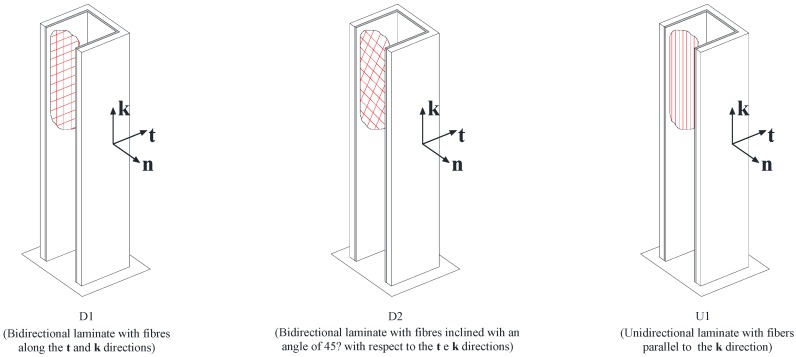
Different types of strengthening under consideration.

The study has been approached by considering three different configurations for fiber arrangements, denoted in the following by “*D1*”, “*D2*” and “*U1*” (Figure 5).

In the first case (“*D1*”)*,* the strengthening is carried out at the inner side of the RC member using a bidirectional FRP wrapping, whose fibers are aligned to the axes **t** and **k** of the local reference system, as indicated in the same figure.

In the second case (“*D2*”), a bidirectional FRP wrapping is also used; unlike the first case, however, the fiber directions of the composite are rotated in the planes (**t**, **k**) by an angle equal to 45° with respect to the previous case.

In the last case (“*U1*”)*,* the strengthening is made only of unidirectional FRP wrapping with the fibers aligned to the direction of the **k** axis.

The three configurations indicated in Figure 5 exemplify three different types of strengthening, which guarantee different levels of effectiveness and will be discussed in the following.

The mechanical properties of the constituent materials assumed in the analysis are summarized in Table 1.

**Table 1:** Mechanical characteristics of the materials.

Material	Young modulus [N/mm^2^]	Shear modulus [N/mm^2^]
Concrete (C20/25)	*E*_c_ = 28,460	*G*_c_ = 14,230
Carbon fibres	*E*_f_ = 235,000	–
Epoxy resin	*E*_r_ = 3800	*G*_r_ = 1380

Moreover, the Poisson’s ratio of the concrete has been assumed equal to zero.

The mechanical properties of the composite strengthening have been obtained from the properties of the fibers (*f*) and the resin (*r*) through the well-known homogenization “*rule of mixture*”.

The laminates considered in this study include bi-directional layers (case “*D1*” and “*D2*”) as well as unidirectional layers (case “*U1*”). The values of the fiber volume fractions are reported in Table 2.

**Table 2:** Volumetric fraction of fibers along the local axes.

Axis	D1	D2	U1
**n**	0.00	0.00	0.00
**t**	0.25	0.25	0.00
**k**	0.25	0.25	0.50

The compliance coefficient (5–7) have been established considering an appropriate secant value related to the cohesive interfacial law suggested in [18] for simulating the bonding of FRP to concrete.

## 4. Numerical Results

The analysis has highlighted the regime of the torsional moments within both the concrete wall-system and the composite overlay.

**Figure 6:**
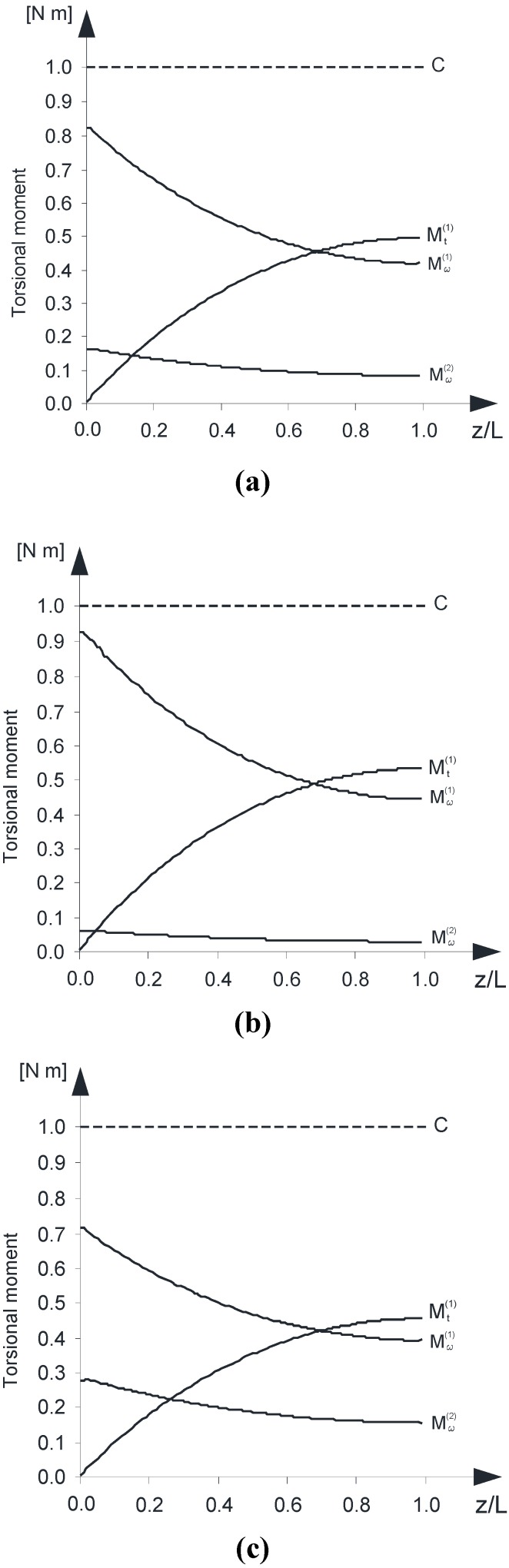
(**a**) Saint Venant and warping torsional moments *versus* z/L—(FRP type: “D1”); (**b**) Saint Venant and warping torsional moments *versus* z/L—(FRP type: “D2”); (**c**) Saint Venant and warping torsional moments *versus* z/L—(FRP type: “U1”).

In particular, Figures 6 a–c show the diagrams of the Saint Venant torsional moment ($M_{\text{t}}^{\left(1\right)}$) and the warping torsional moment ($M_{\omega }^{\left(1\right)}$) for the beam B^(1)^ as well as the warping torsional moment ($M_{\omega }^{\left(2\right)}$) within the beam B^(2)^.

As expected, the beam B^(2)^ does not exhibit appreciable values of the Saint Venant torsional moment, due to the low thickness of the composite.

The authors underline that the regime of the warping torsional moment ($M_{\omega }^{\left(2\right)}$) exhibited by the beam B^(2)^ is almost completely extinguished if is removed the hypothesis of zero warping displacements at the bottom end $\Sigma_{\left(0\right)}$.

**Figure 7:**
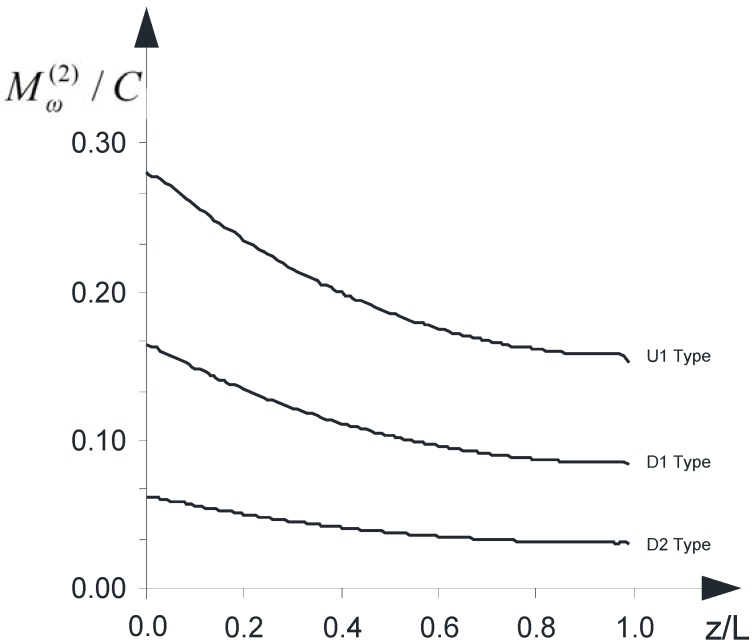
The partition coefficient $M_{\omega }^{\left(2\right)}/C$ *versus* z/L.

Figure 7 shows the effectiveness of the strengthening varying with regard to what type of intervention is considered: “*D1*”, “*D2*” or “*U1*”. The following partition coefficient has evaluated along the **k** axis: $M_{\omega }^{\left(2\right)}/C$. It emerges that the type “*U1*” makes it possible to achieve the greatest benefits in terms of reduction of internal torsional moment ($M_{t}^{\left(1\right)}$ + $M_{\omega }^{\left(1\right)}$) within the concrete wall-system. The minimum and maximum values are summarized in Table 3.

**Table 3:** Minimum and maximum value of the coefficient $M_{\omega }^{\left(2\right)}/C$.

	D1	D2	U1
min	0.08	0.03	0.15
max	0.16	0.06	0.28

**Figure 8:**
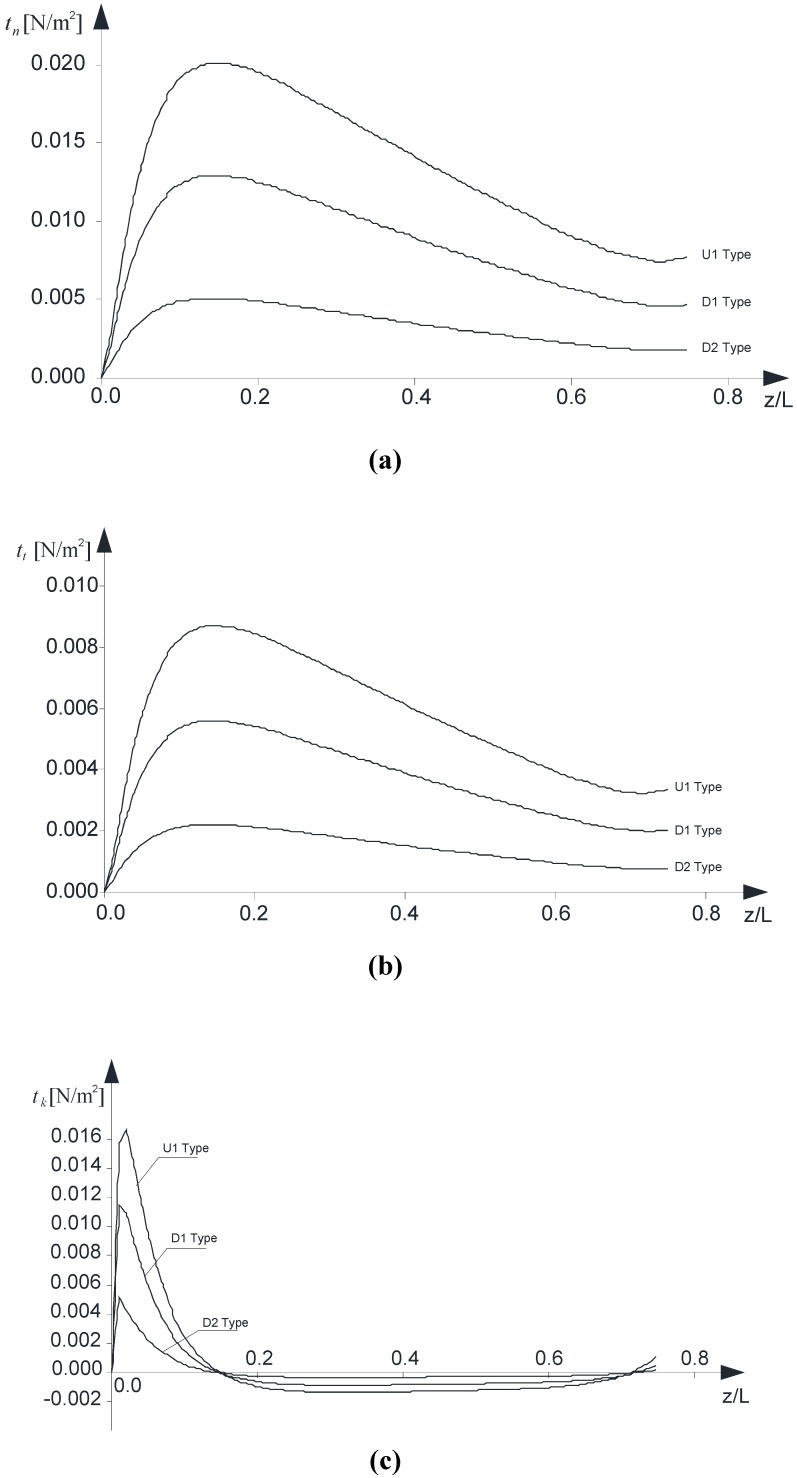
(**a**) Normal interactions, *t**_n_***, *versus* z/L; (**b**) Tangential interactions along the **t** direction, *t**_t_***, *versus* z/L; (**c**) Tangential interactions along the **k** direction, *t**_k_***, *versus* z/L.

The peak values of interfacial interactions in the cross-sections *versus* the normalized axial coordinate are also investigated (Figure 8 a–c). In line with what was discussed in sub-section 2.2, the interactions related to the local axes **n, t** and **k** are presented in order.

It should be noted that the plots are limited to the following range: 0≤z/L≤0.75 as *z* approaches the top end Σ_(*l*)_ (0.75≤z/L), due to the well-known singularity of the contact between two elastic bodies, the result obtained via the interface model adopted are no longer reliable.

Although refined models have been developed which account, at the cut-off zone, for a soft transition of the interfacial interactions from zero to the regime value [19,20], the authors remark that the simplified interface model used is appropriate if the aim is just to underline the feasibility of such a structural application, mainly depending on the warping constraint at the bottom end Σ_(0)_.

**Figure 9:**
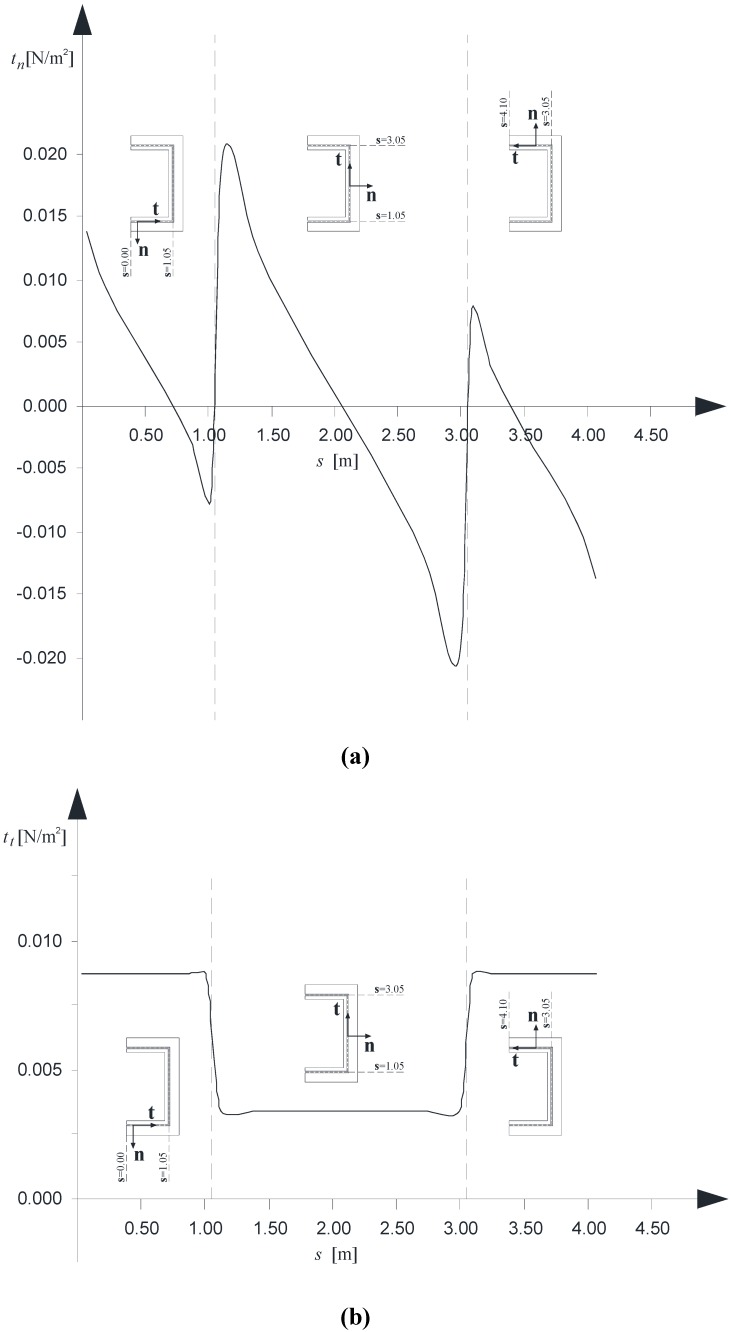

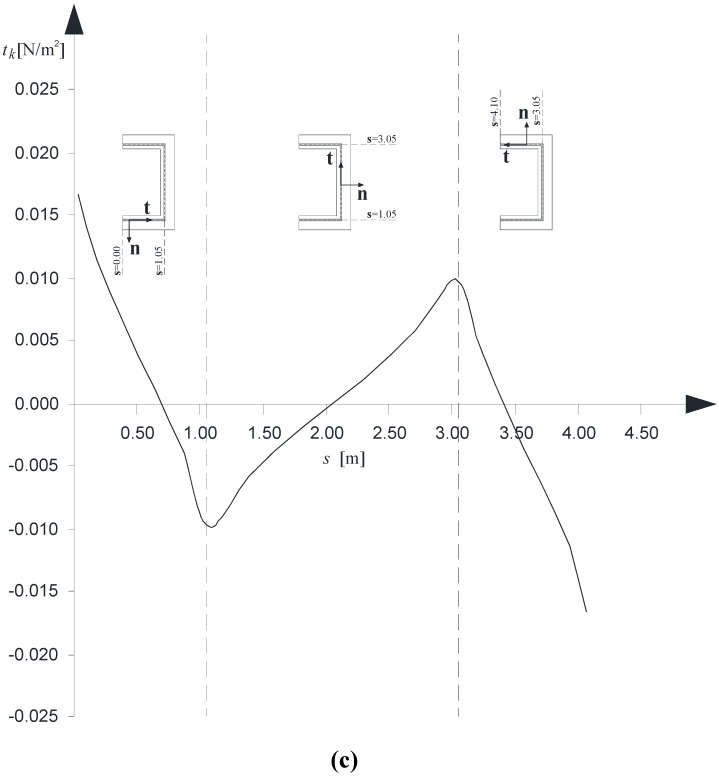
(**a**) Normal interactions, *t**_n_***, *versus* the *s* coordinate; (**b**) Tangential interactions along the **t** direction, *t**_t_***, *versus* the *s* coordinate; (**c**) Tangential interactions along the **k** direction, *t**_k_***, *versus* the *s* coordinate.

Finally, Figures 9 a–c present, in order, the normal, transverse and longitudinal interactions along the contact line κ between the B^(1)^ and B^(2)^. The three diagrams refer to case “U1” and have been evaluated at the cross-sections where the peak value is reached, as it can be argued by the previous Figures 8 a–c.

## 5. Conclusion 

The numerical simulations carried out assess the feasibility of the structural rehabilitation of concrete wall-systems with open cross-sections by using composite laminates in the perspective of increasing their torsional strength, via a strategy which make it possible that the warping moment arises within the composite reinforcement.

The analysis, although based upon a simplified interface model and a simplified constitutive law for the R.C. member, has pointed out that, starting from composites made of the same density of fiber, the arrangement of fibers plays a significant role on the effectiveness of the strengthening intervention.

In detail, the configuration characterized by a greater density of the longitudinal fibers ensures a more efficient partition of internal stresses, thus reducing the torsional moment within the concrete member.

Finally, the analysis also highlights the non-uniform distribution of the interactions between the RC element and the FRP in the cross section and along the beam axis.
